# Supplementary material for: Baseline seabed habitat and biotope mapping for a proposed marine reserve
Source: PeerJ. 2015 Dec 10;3:e1446. doi: 10.7717/peerj.1446 (PMC4690383; doi:10.7717/peerj.1446)
Supplement: Table S1 — Confident assessment of the current study using the Mapping European Seabed Habitats (MESH) project. [file peerj-03-1446-s002.docx]

| Remote Technique | 2 |
| --- | --- |
| Remote Coverage | 1 |
| Remote Positioning | 3 |
| Remote Stds Applied | 2 |
| Remote Vintage | 3 |
| BGT Technique | 1 |
| PGT Technique | 1 |
| GT Positioning | 3 |
| GT Density | 3 |
| GT Stds Applied | 2 |
| GTVintage | 3 |
| GTInterpretation | 2 |
| RemoteInterpretation | 3 |
| DetailLevel | 2 |
| MapAccuracy | 1 |
| Remote score | 73 |
| GT score | 68 |
| Interpretation score | 66 |
| Overall score | 69 |
